# Estimating the size of the MSM populations for 38 European countries by calculating the survey-surveillance discrepancies (SSD) between self-reported new HIV diagnoses from the European MSM internet survey (EMIS) and surveillance-reported HIV diagnoses among MSM in 2009

**DOI:** 10.1186/1471-2458-13-919

**Published:** 2013-10-03

**Authors:** Ulrich Marcus, Ford Hickson, Peter Weatherburn, Axel J Schmidt

**Affiliations:** 1 Department of Infectious Disease Epidemiology, Robert Koch Institute, P.O. Box 650261, 13302 Berlin, Germany; 2 Sigma Research, London School of Hygiene and Tropical Medicine, London, UK

**Keywords:** Men having sex with men, MSM population size, Internet survey, Surveillance systems, HIV diagnosis, Europe

## Abstract

**Background:**

Comparison of rates of newly diagnosed HIV infections among MSM across countries is challenging for a variety of reasons, including the unknown size of MSM populations. In this paper we propose a method of triangulating surveillance data with data collected in a pan-European MSM Internet Survey (EMIS) to estimate the sizes of the national MSM populations and the rates at which HIV is being diagnosed amongst them by calculating survey-surveillance discrepancies (SSD) as a measure of selection biases of survey participants.

**Methods:**

In 2010, the first EMIS collected self-reported data on HIV diagnoses among more than 180,000 MSM in 38 countries of Europe. These data were compared with data from national HIV surveillance systems to explore possible sampling and reporting biases in the two approaches. The Survey-Surveillance Discrepancy (SSD) represents the ratio of survey members diagnosed in 2009 (HIVsvy) to total survey members (Nsvy), divided by the ratio of surveillance reports of diagnoses in 2009 (HIVpop) to the estimated total MSM population (Npop). As differences in household internet access may be a key component of survey selection biases, we analysed the relationship between household internet access and SSD in countries conducting consecutive MSM internet surveys at different time points with increasing levels of internet access. The empirically defined SSD was used to calculate the respective MSM population sizes (Npop), using the formula Npop = HIVpop*Nsvy*SSD/HIVsvy.

**Results:**

Survey-surveillance discrepancies for consecutive MSM internet surveys between 2003 and 2010 with different levels of household internet access were best described by a potential equation, with high SSD at low internet access, declining to a level around 2 with broad access. The lowest SSD was calculated for the Netherlands with 1.8, the highest for Moldova with 9.0. Taking the best available estimate for surveillance reports of HIV diagnoses among MSM in 2009 (HIVpop), the relative MSM population sizes were between 0.03% and 5.6% of the adult male population aged 15–64. The correlation between recently diagnosed (2009) HIV in EMIS participants and HIV diagnosed among MSM in 2009 as reported in the national surveillance systems was very high (R^2^ = 0.88) when using the calculated MSM population size.

**Conclusions:**

Npop and HIVpop were unreliably low for several countries. We discuss and identify possible measurement errors for countries with calculated MSM population sizes above 3% and below 1% of the adult male population. In most cases the number of new HIV diagnoses in MSM in the surveillance system appears too low. In some cases, measurement errors may be due to small EMIS sample sizes. It must be assumed that the SSD is modified by country-specific factors.

Comparison of community-based survey data with surveillance data suggests only minor sampling biases in the former that – except for a few countries - do not seriously distort inter-country comparability, despite large variations in participation rates across countries. Internet surveys are useful complements to national surveillance systems, highlighting deficiencies and allowing estimates of the range of newly diagnosed infections among MSM in countries where surveillance systems fail to accurately provide such data.

## Background

International comparisons of the incidence of newly diagnosed HIV infections among men who have sex with men (MSM) are challenging for a variety of reasons. These include: differences in national infectious disease surveillance systems (e.g. physician-based or laboratory-based reporting); difficulties in measuring the sexual orientation of patients and the challenges of collecting this data at the national level; and cultural, historical, and political differences between countries that affect the degree of openness about sexual preferences and practices during communication between care providers and patients. On the other hand, self-reported data from anonymous convenience samples have other challenges due to selection and self-selection biases introduced through the sampling method. Relevant to both measurement strategies is the unknown size of the MSM population (which critically depends on the definition of MSM [1]), while the relative size of this population (the proportion of the adult male population who have sex with men) may be different between countries and may change over time in response to social, economic, political and cultural developments.

We have previously demonstrated that data from community based internet surveys among MSM can be used to estimate regional population distributions and to calculate the absolute size of regional MSM populations. Together with national surveillance data, MSM-specific incidence rates for reportable sexually transmitted infections can be estimated [2,3].

Participants in open access community-recruited surveys - such as the European MSM Internet Survey (EMIS) conducted in 2010 - are unlikely to be representative of the MSM population of a country. Surveys addressing sexual behaviour, sexually transmitted infections and HIV can be expected to have a self-selection bias towards men with a greater interest in sex and HIV, and of gay community attached MSM, particularly those diagnosed with HIV [4]. A comparison between HIV prevalence estimates derived from modelling approaches based on surveillance data and self-reported HIV prevalence from EMIS suggested that HIV positive men might be approximately twice more likely to participate in EMIS than in a hypothetical “representative” MSM sample [5]. However, this comparison is based on countries with comparable internet accessibility, comparable legal status of homosexuality and social acceptance of same sex partnerships. Whether EMIS participants from countries with lower internet accessibility, and higher levels of social, legal and political stigmatization of homosexuality are to an equal degree representative for the MSM population of their countries is unknown. It is also unknown whether – given adequate interviewing techniques - similar proportions of the adult male population in such countries would report same sex sexual behaviour and partners in representative population surveys, because typically these questions are not addressed in population surveys in such countries.

Analysis of EMIS participation rates by age group on national level has shown that participation reflects accessibility of the target group on MSM websites, which are instrumental for recruitment of survey participants [6]. This suggests that variability in participation rates to community-based internet surveys by country is likely also related to external factors such as access to the internet, and popularity of internet dating and contact sites for MSM in different countries. Whether and to what degree relative sizes of the respective MSM populations differ, and which other factors impact on internet survey participation rates has not been analysed before.

To address these questions, the number of newly diagnosed and reported HIV infections in MSM in the year 2009 derived from national surveillance systems in Europe were compared with self-reported survey derived data on newly diagnosed HIV infections in survey participants of EMIS who were tested for HIV in 2009.

## Methods

### Survey (EMIS) derived data

A detailed description of the survey methods are published elsewhere [7,8]. Briefly, six Associated Partners (APs) recruited another 77 Collaborating Partners (CPs) from academia, public health and civil society across 35 countries.

Five international MSM dating websites were contracted to send instant messages (IMs) to members in a series of waves. Endorsement of the study by the websites provided community support.

Fieldwork occurred during June-August 2010. Over 184,469 responses were submitted of which 94.4% were eligible. Partners in 38 countries were handed back a national database of 100 or more respondents for national analysis and outputting, while the AP team proceeded on International comparisons among 174,000 respondents in 38 countries.

EMIS was approved by the Research Ethics Committee of the University of Portsmouth, United Kingdom (REC application number 08/09:21).

The proportion of the total adult male population who participated in this survey (*participation rate*) was calculated for 38 countries and varied widely, from 20.2 per 10,000 to 0.8 per 10,000 of the male adult population (see Additional file 1: Table S1 column P). As far as *response rates* per recruiter website could be determined - almost 107,000 out of a total of more than 180,000 respondents were recruited via personalized invitations from two supranational gay websites - differences in response rates were much smaller, between 4 and 14% of those invited to participate.

**Table 1:** **Calculated and estimated data for survey-surveillance discrepancies and MSM population size in 38 countries of Europe (see also Additional file****1****: Table S1)**

**Country code**	SSD calculated by SSD=1.67* [household internet access] ^-0.6	**Npop calculated with SSD**	**Percent of the adult male population (Ntot) represented by Npop (=M)**	**Npop with truncation for outliers <1% and >3%**^**1**^**(suggested “best” estimate)**
AT	2.09	70985	2.51	70985
(BA)	5.79	26044	1.68	26044
BE	***2.19***	**148081**	**4.18**	106336
BG	***3.81***	**15923**	**0.60**	26341
BY	5.09	**9368**	**0.29**	31836
CH	1.93	70229	2.69	70229
(CY)	2.89	6954	2.29	6954
CZ	***2.66***	46321	1.24	46321
DE	***1.99***	655740	2.41	655740
DK	1.88	**88900**	**4.87**	54723
EE	2.31	0		9195
ES	2.50	294028	1.86	294028
FI	2.03	**98663**	**5.57**	53118
FR	***2.22***	**821326**	**3.93**	626948
GR	3.37	102888	2.72	102888
(HR)	2.69	29497	1.95	29497
HU	2.58	53404	1.55	53404
IE	2.20	47697	3.08	46488
IT	2.63	359315	1.81	359315
LT	2.50	17760	1.55	17760
(LU)	1.91	**1749**	**1.05**	1749
LV	2.45	12880	1.65	12880
MD	9.03	15853	1.32	15853
MK	3.48	**1232**	**0.17**	7390
(MT)	2.29	3545	2.49	3545
NL	***1.83***	111072	2.00	166872
NO	1.85	**56459**	3.57	47483
PL	***2.61***	**67482**	**0.50**	134981
PT	2.66	109171	3.05	107328
RO	***3.41***	**2939**	**0.04**	74916
RS	3.62	36944	1.62	36944
RU	3.44	**243384**	**0.53**	461264
SE	1.85	65632	2.16	65632
SI	2.29	**39427**	**5.48**	21591
SK	2.31	**18614**	0.96	19366
TR	3.80	**6890**	**0.03**	232935
UA	6.53	**55547**	**0.36**	154415
UK	2.05	**694617**	3.40	613658

^1^ calculated Npop values below 1% and above 3% of the adult male population are substituted by 1% resp. 3% of the adult male population.

* adjusted for risk re-distribution of cases with unknown transmission risk.

**Bold,** questionable or unreliable values.

***Italics,*** SSD value likely too low (or too high: BE, FR).

Survey participants were asked whether they had ever been tested for HIV, and about the date and result of their last test. From these questions we determined the number of survey participants (not already diagnosed with HIV at the beginning of 2009) who reported to have been tested and diagnosed with HIV in 2009.

In addition, the total size of the national samples was used as a second survey-derived parameter in the analysis.

### Surveillance system-derived data

Newly diagnosed HIV among MSM in 2009: National surveillance data on newly diagnosed HIV infections among MSM in 2009 as submitted to the European Centre for Disease Prevention and Control (ECDC) and reported in the HIV 2009 and HIV 2010 surveillance reports were verified and updated by consulting the nominated contact points for HIV surveillance in respective countries. Contact points from 32 countries responded (with no response from Austria, Belorussia, Bulgaria, Croatia, Malta, and Moldova). Due to unresponsiveness of official surveillance institutions, contact points in Russia and Turkey consisted of EMIS NGO partners. According to information provided by the respective national contact points the data reported to ECDC or published elsewhere were adjusted for reporting delays. Cases with unknown transmission risk were proportionally redistributed to transmission groups, and incomplete national coverage of surveillance data (HIV data reported from Spain and Italy did not cover the whole country in 2009) was taken into account for the calculation of the notification rates (see Additional file 1: Table S1, column H).

### Data from national and European statistics

The total size and age distribution of the adult male population (15–64 years) in the European countries included in the analysis were taken from Eurostat, and from national statistics for countries outside the European Union/European Free Trade Association (EU/EFTA) [9].

The proportion of households with internet access was also taken from Eurostat or national statistics [10,11].

### Calculation of the survey-surveillance discrepancy (SSD) and estimating the SSD-range and the total MSM population size

The following formula describes the survey-surveillance discrepancy (SSD)**:**

$$\begin{array}{l} \mathrm{SSD}=\frac{\mathrm{HI}{\mathrm{HIV}}_{\text{svy}}}{N_{\text{svy}}}÷\frac{\text{HI}V_{\text{pop}}}{N_{\text{pop}}}=\frac{\text{HI}V_{\text{svy}}}{N_{\text{svy}}}÷\frac{\text{HI}V_{\text{pop}}}{M•N_{\text{tot}}}\\ \;=\frac{\text{HI}V_{\text{svy}}•M•N_{\text{tot}}}{N_{\text{svy}}•\text{HI}V_{\text{pop}}} \end{array}$$

where

N_svy_ is the sample size in a national survey;

HIV_svy_ is the number of those survey participants diagnosed with HIV in 2009;

HIV_pop_ is the number of HIV cases diagnosed among MSM reported to the countries surveillance system in 2009 (adjusted as described above);

N_pop_ is the estimated total size of the male MSM population, which comprises two measures;

N_tot_, the number of men in the country and

M, the proportion who have sex with men.

For each country, the SSD represents the ratio of the incidence of recent diagnoses among survey members living in a country to the incidence of recent diagnoses in that country’s national surveillance system.

**Figure 1:**
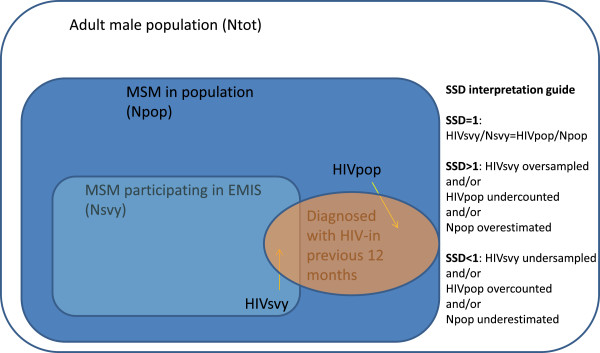
Graphical illustration of Survey Surveillance Discrepancies (SSD) and SSD interpretation guide.

The interpretation of SSD values is illustrated in Figure 1. It must be emphasized that the MSM population as defined by this equation is the population which contributes to the HIV epidemic among MSM. MSM individuals or subgroups which do not contribute to the HIV epidemic because they are not effectively connected would not be included in this estimate. Insofar as epidemiological connectedness is part of the definition, the size of the population may be different from estimates derived from population based surveys asking for lifetime prevalence of same sex partners.

Four of the six parameters from the SSD equation are available to us for most countries: the two survey-derived parameters were measured; surveillance-systems provide the best estimates for the number of new HIV diagnoses among MSM; and national statistics provide the number of men in the countries.

The proportion of men who are homosexually active has been measured in only few European countries. For the purpose of this analysis we took the proportion of men homosexually active (defined as having had at least one sexual contact with a man within the previous 12–24 months) to be between 1% and 3% of the adult male population, based on published results from general population surveys addressing this question [12,13].

We hypothesized that the main bias in survey participation is due to variance in household internet access. It has been proposed by other internet researchers that the internet first attracts the most socially and sexually active MSM [14]. With broader availability of internet access in the population a “regression to the mean” occurs. Thus, a linear relationship between household internet access and SSD could not be expected.

To test this hypothesis, we looked for data from repeated MSM internet surveys from the same countries to calculate the respective SSD values. If the surveys included different time-points with increasing levels of household internet access, and if we assume that over the time period in which these surveys were conducted the size of the MSM population didn’t change substantially, an SSD can be calculated for the respective consecutive surveys (see Additional file 2).

**Figure 2:**
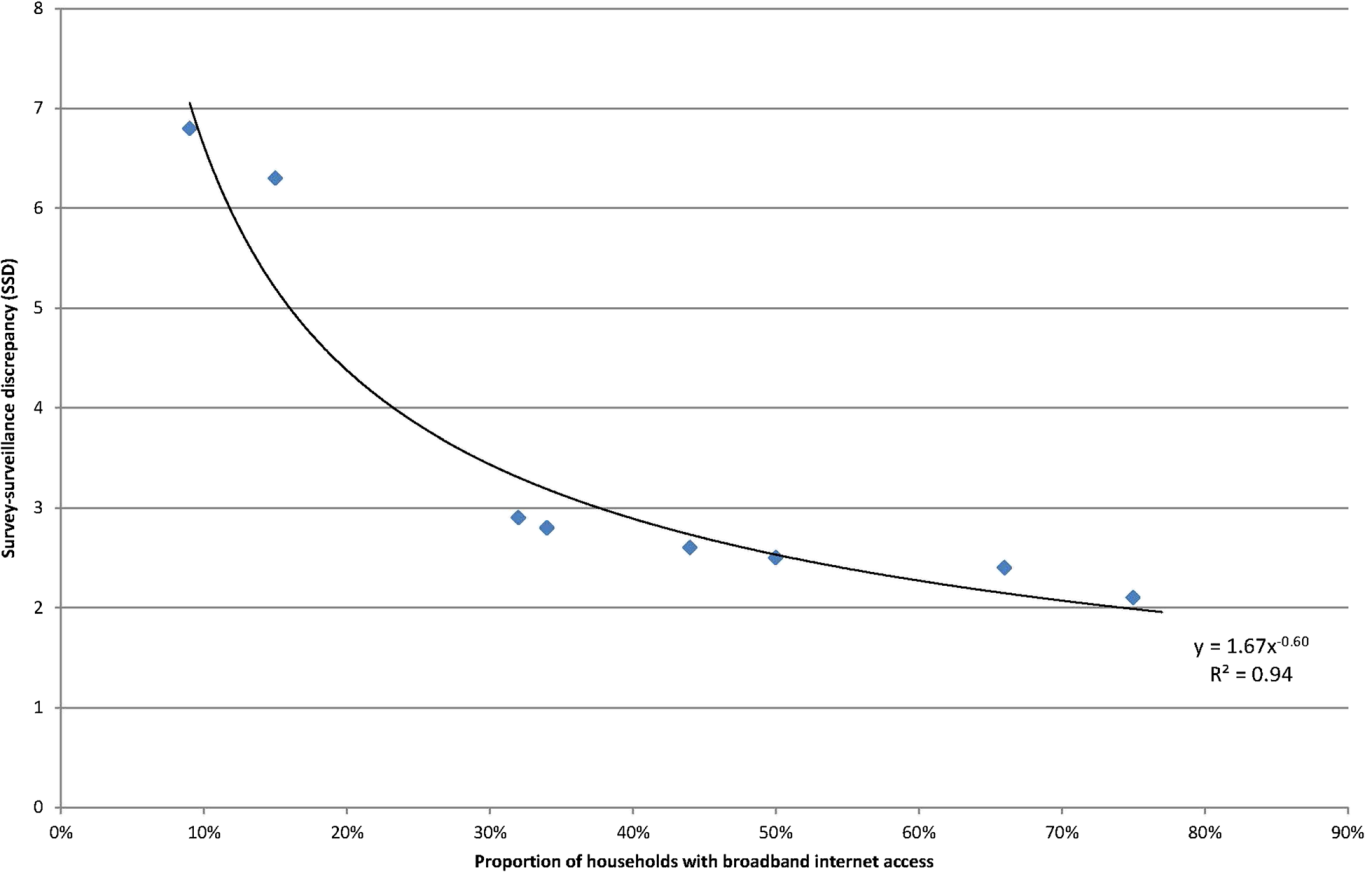
Association between proportion of households with broadband internet access and calculated survey-surveillance discrepancy based on countries with repeated internet-based behavioural surveys among MSM between 2003 and 2010.

When different SSD values were calculated at the same household internet access level, the median was determined. Obvious outlier values are likely explained by targeted offline promotion of internet surveys (SSD values increased) or mis-interpretable wording of respective questions (values too low) and were disregarded. The mathematical function which best described the observed correlation between SSD and household internet access from the countries with repeated surveys (SSD = 1.67*[national household internet access]^-0.6^ - see Additional file 2 and Figure 2) was then used to calculate an SSD for all 38 EMIS countries. The resulting SSD was further used to estimate the total MSM population size for all 38 countries (Npop = HIVpop*Nsvy*SSD/HIVsvy).

For all countries with an MSM population size estimate outside of the range 1% to 3% of the adult male population, the validity of the other survey- and surveillance-derived parameters is discussed.

### Estimating a plausible range for the number of MSM newly diagnosed with HIV in 2009 for countries with missing or unreliable surveillance system-derived data

For countries reporting zero HIV diagnoses among MSM in 2009 (Estonia) in their surveillance system, countries not reporting HIV diagnoses among MSM at all (Austria), and countries reporting implausibly low numbers of MSM (resulting in MSM population estimates well below 1% of the male adult population: Bulgaria, Belarus, Romania, Russia, Turkey, and Ukraine), we estimated the minimum number of MSM expected to have been diagnosed with HIV in 2009 for an assumed MSM population size of at least 1% (resp. 2% for Estonia – comparable with Latvia and Lithuania - and 3% for Austria – comparable with Germany and Switzerland) given the SSD estimated on the proportion of households with internet access (see Additional file 1: Table S1, column I). Poland, Russia, Belarus, and Turkey are countries reporting a high proportion of cases with unknown routes of transmission [15,16].

For countries, in which the EMIS sample didn’t include somebody diagnosed with HIV in 2009 (Bosnia-Herzegovina, Cyprus, Estonia, Malta, Moldova) we calculated, how many participants diagnosed with HIV in 2009 would have been expected to participate in EMIS to achieve a number compatible with the SSD and an MSM population size of 1% (Bosnia-Herzegovina, Moldova) resp. 2% (Cyprus, Estonia, Malta) of the male adult population.

## Results

### Establishing the relationship between SSD and household internet access

Repeated internet surveys among MSM collecting information on HIV diagnosed in survey participants in the year before the survey were identified from five Western and Central European countries: Germany [17], France, the Netherlands, Switzerland [18], and the UK [19]. The surveys were conducted in the years 2003 to 2010. Recruitment strategies were not fully comparable for these surveys, since different countries and surveys used different mixes of online and offline promotion. The German surveys were the ones which most exclusively used online promotion, similar to the predominant mode of promotion for EMIS. In addition, how the outcome parameters - the number of respondents tested and diagnosed with HIV (ever and in the previous 12 months) - were queried differed between and even within countries, so that some surveys could not be included in the SSD calculation (e.g. the Dutch surveys). Additional file 2 presents (1) the proportion of households with internet access in the years 2003 – 2010 in Germany, France, the UK, and Switzerland, (2) the number of internet survey participants, (3) the number of survey participants diagnosed with HIV in the year before the survey, and (4) the number of HIV diagnoses among MSM reported in the surveillance system in the respective year. For simplification, in all these countries (5) the absolute size of the MSM population was assumed to be 3% of the adult male population (aged 15–64). The SSD for the different surveys is presented in the table Observed SSD in the Additional file 2.

A clear correlation of declining SSD with increasing household internet access was observed in all countries. Figure 2 [SSD empirical] presents the correlation between level of internet access and SSD from the respective internet surveys. Additional file 1: Table S1 presents the levels of household internet access for the 38 EMIS countries (column C) and the respective SSD values derived from the empirical correlation (column J, see also Table 1) between internet access and SSD observed in the countries with repeated surveys.

The SSD values calculated on the basis of household internet access distinguish well between the nine larger geographical regions that were used to group the 38 EMIS countries (Table 2).

**Table 2:** Mean SSD values for EMIS sub-regions

**EMIS sub-region**	**Countries**	**Mean SSD**
North-western Europe	DK; FI; NO; SE	1.9
Central West Europe	AT; CH; DE; LU	2.0
Western Europe	BE; FR; IE; NL; UK	2.1
North-eastern Europe	EE; LT; LV	2.4
Central East Europe	CZ; HU; PL; SI; SK	2.5
South-western Europe	ES; GR; IT; PT	2.8
South-eastern Europe (EU)	BG; CY; MT; RO	3.1
South-eastern Europe (non-EU)	BA; HR; MK; RS; TR	3.9
Eastern Europe	BY; MD; RU; UA	6.0

### Data measured in EMIS and reported in the national surveillance systems

EMIS recruited more than 100 eligible participants in 38 European countries. The characteristics of participants are reported in the EMIS 2010 Report [8].

Table 1 presents the estimated SSD based on the formula SSD = 1.67*[national household internet access]^-0.6^, an estimate of the total size of the MSM population based on the formula N_pop_ = HIV_pop_*N_svy_*SSD/HIV_svy_, and the proportion of the total adult male population (M) that would be estimated to be MSM based on this N_pop_ estimate. Additional EMIS-measured and surveillance data necessary to calculate these estimates, and other reported data such as the proportion of households with internet access in the 38 countries with EMIS sample sizes exceeding 100 participants [column C] are presented in Additional file 1: Table S1.

In five countries with small absolute sample sizes - Bosnia-Herzegovina, Cyprus, Estonia, Malta, and Moldova - no EMIS-participants reported to have been newly diagnosed with HIV in 2009. In 13 countries the number of EMIS participants newly diagnosed with HIV in 2009 was below 10, making the use of these values for further calculations less reliable.

Using the data listed in Additional file 1: Table S1 to estimate the total size of the MSM population resulted in populations significantly smaller than 1% of the adult male population for Belarus, Bulgaria, Macedonia, Moldova, Poland, Romania, Russia, Turkey, and Ukraine. For Slovakia the estimated population size was slightly lower than 1% (Table 1).

The estimated MSM population size was larger than 3% for Belgium, Denmark, Finland, France, Norway, Slovenia, and the UK. In all these countries the ratio of EMIS participants reporting to have been diagnosed in 2009 to the number of MSM diagnosed in 2009 and reported within the national surveillance system was comparatively low (<0.07) (Additional file 1: Table S1, column N).

**Figure 3:**
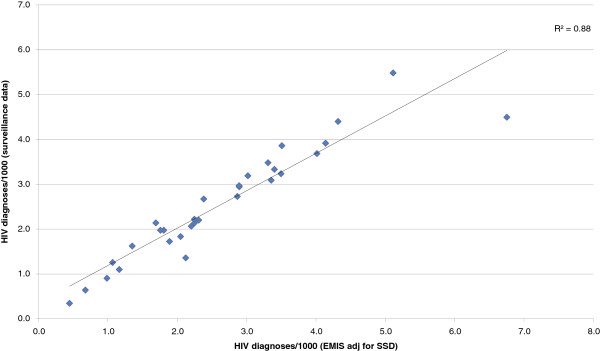
**Association between number of newly diagnosed HIV infections among EMIS respondents in 2009 (after SSD adjustment) and notification rate of HIV infections among MSM per 1000 MSM (MSM population size estimate based on Table **1**, last column).**

Figure 3 [correlation] shows the correlation between newly diagnosed HIV as reported by national surveillance systems per 1,000 MSM (using the estimates from column H/I and column M in Additional file 1: Table S1) and the proportion of survey participants who reported having been diagnosed with HIV in 2009 after SSD adjustment. The correlation coefficient is very high with an R^2^ = 0.88. Without the outliers Poland and the Netherlands, for which the calculated SSD may be too low (as discussed below), the correlation coefficient would increase to R^2^ = 0.95. The countries with no EMIS participants diagnosed with HIV in 2009 are excluded from this analysis.

### Estimation of the number of newly diagnosed HIV among MSM in 2009 for countries not reporting these data or reporting unfeasibly low numbers

When we take SSD values based on the relation between household internet access and SSD for countries without HIV surveillance data on infections diagnosed in MSM or with unreliable data and assume that the MSM population size is at least 1% of the adult male population, the lower limit of infections diagnosed in MSM can be calculated, using the formula:

$$\mathrm{HI}V_{\mathrm{pop}}=\frac{\mathrm{HI}V_{\mathrm{pop}}}{\mathrm{SSD}}•\frac{N_{\mathrm{pop}}}{N_{\mathrm{svy}}}$$

As shown in Additional file 1: Table S1 [column I], under these assumptions the minimum number of MSM diagnosed in 2009 in Belarus, Russia, and Ukraine would be estimated to be at least 4–5 times larger than officially reported, in Turkey even 40 times larger.

## Discussion

We propose the calculation of the Survey-Surveillance Discrepancy (SSD) as a method to compare self-reported data on newly diagnosed HIV between different countries, and with surveillance system-derived data. Internet access rates are one important factor to take into account when comparing self-reported data from internet samples, as we can demonstrate from previous internet surveys conducted in different countries of Western Europe between 2003 and 2010. However, already these data from repeated surveys suggest that survey promotion strategies may also have an impact on SSD. The general promotion strategy for EMIS was the same in all countries, with some country-by-country variation of offline promotion activities and of the proportion of recruitment via individualized invitation messages. These differences were not adequately captured by our simplifying assumption that household internet access is the principal determining factor of survey-surveillance discrepancy. Although calculating the SSD based on an empirically observed relation between access rates and SSD clearly distinguishes between geographically defined groups of countries with similar political, historical, cultural characteristics, additional variability of the SSD within these country groups is likely.

A limitation of this approach is the lack of respective longitudinal data from countries in Eastern and Central Europe (WHO classification). Thus it is not possible to rule out that in addition to household internet access, different factors than in Western European countries modify the SSD in those countries.

If we look at the results of SSD and population size calculations for individual countries (see Table 1, column Npop calculated with SSD), the estimated MSM population size remains well below 1% of the adult male population for eight countries – Bulgaria, Belarus, Macedonia, Poland, Romania, Russia, Turkey, and Ukraine – given the calculated SSD. For these countries the ratio of EMIS participants reporting to have been diagnosed in 2009 to the number of MSM diagnosed in 2009 and reported within the national surveillance system was larger than 0.2 (Additional file 1: Table S1, column N). This means, more than 1 in 5 newly diagnosed MSM in these countries would have participated in EMIS, although the respective country samples were among the smallest in EMIS, and didn’t exceed 4 per 10,000 male adults or 4% of the MSM population if we assume that in these countries only 1% of the male adults are MSM. However, after risk re-distribution of cases with unknown transmission risk, in Poland and Russia the ratio dropped to a level comparable with other countries (Additional file 1: Table S1, column O). This strongly suggests that surveillance data are unreliable in terms of transmission risk categorization (or number of reported cases) also in Bulgaria, Belarus, Romania, Turkey, and Ukraine.

When comparing the number of EMIS participants diagnosed with HIV in the years before the survey (i.e. between 2000 and 2010) with HIV diagnoses among MSM reported in the national systems [16], the data for Poland, Romania and Bulgaria suggest a disproportionally high participation of men diagnosed with HIV in 2009. This would be compatible with an SSD higher than calculated from household internet access for these countries, and might be explainable by targeting EMIS survey promotion to MSM recently diagnosed with HIV. However, at least for Romania – even if considering a disproportionate participation of newly diagnosed men in EMIS – the data suggest either considerable underreporting of newly diagnosed HIV or a high level of misclassification of MSM into other transmission groups (the number of men diagnosed with HIV in 2009 in the Romanian EMIS dataset was 2.7-fold the national notification rate of MSM in 2009 – see Additional file 1: Table S1, columns E and G). Misclassification should be taken into account, particularly since in all three countries the level of social discrimination of gay men is high [20]. However, in Romania at least, the high proportion of females in newly diagnosed HIV (41% in 2009) suggests substantial heterosexual transmission. Thus, either cases among MSM were underreported or heterosexual transmission is to a considerable degree accounted for by women having sex with bisexual men.

In Macedonia the high ratio (of EMIS participants diagnosed in 2009 to the reported number of diagnosed MSM in 2009) and the very low estimate for the total MSM population may be a consequence of a low number of cases and the chance event that one of three reported cases may have participated in the survey.

The most likely explanation for the findings in all these countries - except Macedonia - is the underreporting of MSM cases in the national surveillance systems, as was suspected already by others [21,22]. At least in Poland and Romania – possibly also in other countries – there may be additional issues with the SSD factor, which may be higher than calculated based on Internet access due to targeted promotion of EMIS to MSM (recently) diagnosed with HIV. Alternatively it may be that not only the transmission group reporting but also the total number of newly diagnosed and reported infections is too low in these two countries.

In addition to these eight countries, the estimates for the MSM population size for another six countries seem questionably low when compared to other countries from the same sub-region. These countries are the Czech Republic, Luxemburg, the Netherlands, Italy, Spain, and to a lesser degree Germany.

For Italy and Spain, the most likely explanation may be errors in the calculation of notification rates of newly diagnosed infections among MSM. In both countries the reporting system does not cover the whole country and misses some regions (although we explicitly tried to take this into account when calculating national notification rates). In addition, transmission risk group assignment may underestimate MSM and overestimate heterosexual cases due to physicians not asking about and patients not reporting sexual preferences. While this is true for all countries, it may be truer for countries in which same sex behaviours are more stigmatized. According to data collected in EMIS [8] this could be the case for Italy, but less so in Spain. In Luxemburg, the problematic parameter may be the number of EMIS participants living in the country who reported having been diagnosed with HIV in 2009. A major proportion of the HIV positive survey participants from Luxemburg may not have been diagnosed in Luxemburg but in the surrounding countries (e.g. Belgium, Germany, or France) or the countries of origin. The proportion of MSM with a migration history is very high in Luxemburg [8](~50%), and more than 90% of men diagnosed with HIV whose current country of residence is Luxemburg report having had sex in other countries in the previous 12 months. If this hypothesis is correct, the calculated MSM population size would increase.

For Germany and the Netherlands, MSM population sizes closer to 3% of the adult male population, and for the Czech Republic, closer to 2% would be expected. As long as the surveillance data are assumed to be correct (it could be argued that adjustments for the number of newly diagnosed MSM reported in the surveillance system are too low), the SSD factor would have to be higher in those countries than expected from household internet access calculation. For the Netherlands, there is indeed some indication for an increased selection bias, and thus an increased SSD value [6], possibly attributable to the yearly internet surveys in this country, which may result in survey fatigue affecting HIV-uninfected and untested men disproportionally more than men diagnosed with HIV. Germany may be a similar case, because internet surveys addressing MSM have been increasingly launched in recent years.

On the other hand, as shown in Table 1, given the calculated SSD the estimated MSM population size exceeds 3% of the adult male population in three of four Scandinavian countries, Belgium, France, the UK, and Slovenia. For the three Scandinavian countries Denmark, Finland, and Norway, as for Slovenia, exceeding the 3% range may just be a chance event due to the small sample sizes. With one or two more EMIS participants diagnosed with HIV in 2009 in each of those countries the MSM population estimates for these countries would be below 3%, within the expected range. Particularly, among Danish and Finnish EMIS respondents only four and two respondents respectively reported having been diagnosed in 2009, while the mean number for men diagnosed in the previous four years were 9.5 and 6.3, and the surveillance data from Denmark and Finland reported no corresponding decline in the numbers of new HIV diagnoses among MSM during these years.

For Belgium, France, and the UK, there are different possible explanations for this finding: (1) The SSD may be lower than expected in these countries. This would mean that men diagnosed with HIV – for whatever reasons - participated to a lesser extent in EMIS than men from other countries. Such reasons would have to reduce the gap between the willingness of men diagnosed with HIV and men not diagnosed with HIV to participate in EMIS. (2) The adjustments made on national level for the number of diagnosed HIV infections among MSM may be too high, i.e. the real numbers may be closer to the numbers reported to ECDC. (3) The MSM population size is indeed larger than 3% of the adult male population, e.g. due to disproportionate immigration of MSM from other countries, as suggested by Dougan et al. [23].

In the EMIS samples from Bosnia-Herzegovina, Cyprus, Estonia, Moldova, and Malta no respondent reported to have been diagnosed with HIV in 2009. It should be noted that the samples for Bosnia-Herzegovina, Moldova, and Malta were also quite small (<=150). In addition, in Estonia also no case was reported within the national surveillance system. When we tested how many cases of newly diagnosed MSM would be required in the EMIS sample to arrive at a total MSM population of at least 1% of the adult male population (Bosnia-Herzegovina, Moldova) or at a percent-range similar to neighbouring countries (Cyprus, Estonia), the values ranged from 0.1 (Bosnia-Herzegovina) to 1 (Cyprus). Thus, having no men diagnosed with HIV in the year 2009 in the respective country sample would not be unexpected considering the small sample sizes and the low diagnosis numbers. Assuming one man diagnosed with HIV in 2009 had participated in EMIS in Estonia, the number of MSM diagnosed in that year with HIV and not reported as MSM in the national surveillance system would be expected between 5 and 10 (from a total of 243 HIV diagnoses in males in 2009).

Austria reports neither the number of new diagnoses of HIV per year nor the proportion in each transmission group. Assuming a similar size of the MSM population and a similar SSD value in Austria as in the neighbouring countries Germany and Switzerland, the number of newly diagnosed MSM in Austria in 2009 would be estimated at 248 men. In a publication of the Austrian Cohort Study from 2013 a number of 507 new HIV diagnoses in Austria were reported for 2009 [24]. Among them, approximately 150–200 might be estimated to be MSM.

Turkey reported 341 new HIV diagnoses in males and 126 new HIV diagnoses in females in 2009. A heterosexual mode of transmission was reported for 216 cases, sex between males for 2 cases. With the SSD calculated for Turkey based on household internet access, and assuming a proportion of 1% MSM among the adult male population, we would expect approximately 80 MSM among the newly diagnosed cases in 2009 (the number would be lower if the SSD was higher, and higher if the proportion of MSM would be larger).

To summarize, under the assumption that the SSD is mainly (but not exclusively) dependent on the household internet access, we estimated SSD values as a function of household internet access based on earlier repeated internet surveys among MSM from several Western and Central European countries. When MSM population sizes are estimated using these calculated SSD values, differences within and between country groups become obvious, which require further explanation and analysis. These differences may partly reflect the impact of additional factors on the SSD calculation, such as the intrinsic lack of data validity resulting from sample size limitations and uncertain transmission risk attribution for reported national surveillance data. However, even beyond those countries where these additional factors may explain implausible results for MSM population size estimates, there are still some countries with large enough samples and relatively reliable surveillance systems for which the population size estimates result in estimates above the assumed upper limit of 3% MSM in the adult male population. For these countries it must be assumed that either the MSM population size is in fact larger than 3%, or that other factors except household internet access exhibit measurable impacts on the SSD. Such factors would have to equalize the willingness of HIV-positive MSM to participate in EMIS compared to HIV-negative or untested MSM.

After transformation into comparable formats, self-reported incidence of HIV diagnosis in a large internet convenience sample of European MSM correlates strongly with surveillance system reported diagnosis incidence. This argues against any large survey-surveillance discrepancies caused by sampling biases of the survey despite large differences regarding the (relative) sample sizes, and supports a high inter-country comparability of self-reported incidence indicators from this first pan-European internet survey for most of the countries. However, for some countries with low household internet access in East and South-East Europe the survey-surveillance discrepancy factor may be up to double and triple as high as for the other countries, which of course had to be considered when comparing self-reported new HIV diagnosis. Unfortunately, for the same countries the surveillance data regarding MSM are particularly unreliable.

The strong correlation between EMIS derived self-reported HIV diagnosis incidence and surveillance system derived data critically depends on an estimated parameter, the size of the respective MSM populations. The SSD, calculated to assess whether similar parts of the MSM population were reached by the internet survey, is also interdependent with the estimated size of the MSM population: if other parameters remain the same and the estimate for the size of the population increases, the SSD also increases. Unfortunately, for most countries no studies have been conducted to generate empirical data on the size of this population. We would like to emphasize that our definition of the MSM population is strictly behavioural (men who had sex with men in the recent twelve months) and describes a population which is sufficiently and effectively connected to contribute to the HIV epidemic among MSM in a measurable way. Thus our definition would not include men who e.g. would like to have sex with other men, but did not have the opportunity to do so, and it may not cover MSM subpopulations marginalized and isolated due to a lack of gay venues, internet access and other means of communication.

Thus, we assume that low levels of internet access as well as social, cultural, political and economic factors such as restrictive laws, high levels of social discrimination, and the lack of a commercial gay infrastructure can restrict the size of the so defined MSM population. All of these factors prevent people from connecting with each other and reduce their opportunity to acquire sexual partners. However, we would like to emphasize that deliberately using such measures to restrict or reduce the size of MSM populations (as currently attempted by “anti-gay propaganda” laws in Russia) in order to prevent the spread of HIV is a violation of human rights. It will also be counterproductive in the longer run, because it will undermine the capacities of MSM communities to mount an effective response towards the HIV epidemic.

An additional factor we didn’t take into account are the different starting points of the HIV epidemics in Western and Central and Eastern Europe, which result in different age ranges for the (HIV vulnerable) MSM populations. A self-reported recent diagnosis rate of 6.7 per 1000 participants in the German EMIS sample may have to refer to an MSM population comprising 3% of the 15 to 64 year old male population, while a self-reported recent diagnosis rate of 13.3 per 1000 participants in the Polish sample may have to refer to an MSM population comprising 1% of the 15 to 49 year old male population.

Thus, if we compare the incidence of HIV diagnoses in MSM communities between different countries and regions in Europe, we should be aware that the relative size of the denominator populations may be different.

## Conclusion

To conclude, HIV diagnosis incidence measured with self-reports in a large pan-European internet survey recruiting primarily through gay dating websites correlate very well with data from the respective national surveillance systems after making the data formats comparable. Supranational internet surveys may therefore be highly useful complements to national surveillance systems. They may even point out deficiencies and flaws in the national surveillance systems and allow estimations of the range of newly diagnosed infections among MSM in countries whose surveillance systems fail to accurately provide such data.

## Competing interests

The authors declare that they have no competing interests.

## Authors’ contributions

All authors participated in the design of the survey tool. In addition: UM instigated the project, led the study design and drafted the manuscript. FH participated in the study design, constructed the online survey and contributed to the manuscript; PW participated in the study design, co-ordinated the survey promotion and contributed to the manuscript; AJS participated in the study design, coordinated the study and the EMIS Network, and contributed to the manuscript. All authors read and approved the final manuscript.

## Pre-publication history

The pre-publication history for this paper can be accessed here:

http://www.biomedcentral.com/1471-2458/13/919/prepub

## Supplementary Material

Additional file 1: Table S1 Measured and reported data for calculation of survey-surveillance discrepancies and MSM population size in 38 countries of Europe. Click here for file

Additional file 2 Determining the empirical association between household internet access levels and survey-surveillance discrepancies. Click here for file

## The EMIS network consists of associated researchers

Rigmor A. Berg (Norwegian Knowledge Centre for the Health Services, Oslo); Michele Breveglieri (Regione del Veneto, Verona); Laia Ferrer (CEEISCat, Barcelona); Percy Fernández-Davila (CEEISCat, Barcelona); Cinta Folch (CEEISCat, Barcelona); Martina Furegato (Regione del Veneto, Verona); Ford Hickson (Sigma Research, London); Harm J. Hospers (University College Maastricht); Ulrich Marcus (Robert Koch Institute, Berlin); David Reid (Sigma Research, London); Axel J. Schmidt (Robert Koch Institute, Berlin); Todd Sekuler (Robert Koch Institute, Berlin); Peter Weatherburn (Sigma Research, London).

## National collaborating partners of the EMIS network

Aids-Hilfe Wien (Austria); Facultés Universitaires Saint-Louis, Institute of Tropical Medicine, Ex Aequo, Sensoa, Arc-en-ciel (Belgium); Vstrecha (Belarus); National Centre of Infectious and Parasitic Diseases, Queer Bulgaria Foundation (Bulgaria); Charles University, Institute of Sexology (Czech Republic); Research Unit in Behaviour & Social Issues (Cyprus); University of Zagreb, Faculty of Humanities and Social Sciences (Croatia); Statens Serum Institut, Department of Epidemiology, stopaids (Denmark); National Institute for Health Development (Estonia); University of Tampere, Department of Nursing Science, Finnish AIDS council (Finland); Institut de veille sanitaire (InVS), AIDeS, Act UP Paris, Sida Info Service, Le kiosque, The Warning (France); Berlin Social Science Research Center (WZB), Deutsche AIDS-Hilfe (DAH), Federal Centre for Health Education, Cologne (BZgA) (Germany); Positive Voice (Greece); Hungarian Civil Liberties Union, Háttér (Hungary);Gay Men’s Health Service, Health Services Executive (Ireland); University of Bologna, Italian Lesbian and Gay Association (Arcigay), Instituto Superiore di Sanità (National AIDS Unit)(Italy); The Infectiology Center of Latvia, Mozaika (Latvia); Center for Communicable Diseases and AIDS (Lithuania); GenderDoc-M (Moldova); schorer (Netherlands); Norwegian Knowledge Centre for the Health Services, The Norwegian Institute of Public Health (Norway); National AIDS Centre, Lamda Warszawa (Poland); GAT Portugal, University of Porto, Medical School, Inst. of Hygiene and Tropical Med. (Portugal); PSI Romania (Romania);PSI Russia (Russia); Safe Pulse of Youth (Serbia); OZ Odyseus (Slovakia); National Institute of Public Health, SKUC-Magnus, Legebitra, DIH (Slovenia); National Centre of Epidemiology, stopsida, Ministerio de Sanidad, Política Social e Igualdad (Spain); Malmö University, Health and Society, RFSL, National Board of Health and Welfare (Sweden); Institut universitaire de médecine sociale et preventive, Aids-Hilfe Schweiz (Switzerland); Turkish Public Health Association, Siyah Pembe Üçgen İzmir, KAOS-GL, Istanbul-LGBTT (Turkey); Gay Alliance, Nash Mir, LiGA, Nikolaev (Ukraine); City University London, Department for Public Health, Terrence Higgins Trust and the CHAPS partners including GMFA, The Eddystone Trust, Healthy Gay Life, The Lesbian and Gay Foundation, The Metro Centre London, NAM, Trade Sexual Health, Yorkshire, MESMAC (United Kingdom).

## European collaborating partners

International Gay and Lesbian Organization (ILGA); European AIDS Treatment Group (EATG); PlanetRomeo.com; Manhunt and Manhunt Cares.
