# Supplementary material for: Estimating the size of the MSM populations for 38 European countries by calculating the survey-surveillance discrepancies (SSD) between self-reported new HIV diagnoses from the European MSM internet survey (EMIS) and surveillance-reported HIV diagnoses among MSM in 2009
Source: BMC Public Health. 2013 Oct 3;13:919. doi: 10.1186/1471-2458-13-919 (PMC3850943; doi:10.1186/1471-2458-13-919)
Supplement: Additional file 1: Table S1 — Measured and reported data for calculation of survey-surveillance discrepancies and MSM population size in 38 countries of Europe. [file 1471-2458-13-919-S1.pdf]

Additional Table S1: Measured and reported data for calculation of survey-surveillance discrepancies and MSM population size in 38 countries of Europe

A	B	C	D	E	F	G	H	I	J	K	L	M	N	O	P
	country code	proportion of households with Internet access	**Nstu** (sample size)	**HIVstu** (diagnosed HIV+ in 2009)	ECDC reported number of HIV diagnoses among males in 2009	ECDC reported number of HIV diagnoses among MSM in 2009	**HIVpop** Updated national surveillance data on HIV diagnosed among MSM in 2009*	HIVpop required to achieve at least 1% or at most 3% of MSM at given SSD	**SSD** calculated by SSD=1.67* (household internet access)$^{-0.6}$	**Npop** calculated with SSD	percent of the adult male population represented by Npop (=**M**)	**Npop** with truncation for outliers <1% and >3% (suggested "best" estimate)	EMIS participants diagnosed in 2009/ reported number of diagnosed MSM in 2009	EMIS participants diagnosed in 2009/ adjusted number of diagnosed MSM in 2009	EMIS participation rates (number of EMIS participants per 10,000 adult males)
1	**AT**	0.69	4,112	30	n.a.	n.a.	248		2.09	70985	2.51	70985		0.12	14.53
2	**(BA)**	0.13	150	0.1	6	3	3		5.79	26044	1.68	26044	0.03	0.03	0.97
3	**BE**	0.64	4,000	29	749	373	490	370	2.19	148081	4.18	106336	0.08	0.06	11.28
4	**BG**	0.25	1,045	7	133	28	28	52	3.81	15923	0.60	26341	0.25	0.25	3.97
5	**BY**	0.16	368	2	563	10	10	40	5.09	9368	0.29	31836	0.20	0.20	1.16
6	**CH**	0.79	5,045	39	472	249	282		1.93	70229	2.69	70229	0.16	0.14	19.35
7	**(CY)**	0.40	267	1	26	9	9		2.89	6954	2.29	6954	0.11	0.11	8.81
8	**CZ**	0.46	2,437	15	130	106	107		2.66	46321	1.24	46321	0.14	0.14	6.54
9	**DE**	0.75	54,774	365	2404	1646	2200		1.99	655740	2.41	655740	0.22	0.17	20.17
10	**DK**	0.82	1,749	4	179	108	108		1.88	88900	4.87	54723	0.04	0.04	9.59
11	**EE**	0.58	590	1	243	0	0	7	2.31	0	0.00	9195			12.93
12	**ES**	0.51	13,136	136	2326	1217	1217		2.50	294028	1.86	294028	0.11	0.11	8.33
13	**FI**	0.72	2,028	2	110	44	48		2.03	98663	5.57	53118	0.05	0.04	11.45
14	**FR**	0.62	11,197	75	3566	1363	2480	2000	2.22	821326	3.93	626948	0.06	0.03	5.36
15	**GR**	0.31	2,964	34	484	266	350		3.37	102888	2.72	102888	0.13	0.10	7.83
16	**(HR)**	0.45	523	2	48	42	42		2.69	29497	1.95	29497	0.05	0.05	3.46
17	**HU**	0.48	2,069	12	107	87	120		2.58	53404	1.55	53404	0.14	0.10	5.99
18	**IE**	0.63	2,196	14	258	138	138		2.20	47697	3.08	46488	0.10	0.10	14.17
19	**IT**	0.47	15,915	120	1906	776	1030		2.63	359315	1.81	359315	0.15	0.12	8.04
20	**LT**	0.51	591	1	131	9	12		2.50	17760	1.55	17760	0.11	0.08	5.15
21	**(LU)**	0.80	279	7	37	23	23		1.91	1749	1.05	1749	0.30	0.30	16.71
22	**LV**	0.53	701	2	170	15	15		2.45	12880	1.65	12880	0.13	0.13	9.00
23	**MD**	0.06	117	0.8	400	12	12	15	9.03	15853	1.32	15853	0.07	0.07	0.97
24	**MK**	0.29	118	1	6	3	3		3.48	1232	0.17	7390	0.33	0.33	1.60
25	**(MT)**	0.59	116	0.3	10	4	4		2.29	3545	2.49	3545	0.08	0.08	8.15
26	**NL**	0.86	3,810	47	930	725	750		1.83	111072	2.00	166872	0.06	0.06	6.85
27	**NO**	0.84	2,100	6	183	87	87		1.85	56459	3.57	47483	0.07	0.07	13.27
28	**PL**	0.48	2,776	37	729	72	345	740	2.61	67482	0.50	134981	0.51	0.11	2.06
29	**PT**	0.46	5,193	40	1080	308	316		2.66	109171	3.05	107328	0.13	0.13	14.52
30	**RO**	0.30	2,338	19	87	7	7	200	3.41	2939	0.04	74916	2.71	2.71	3.12
31	**RS**	0.28	1,108	9	121	83	83		3.62	36944	1.62	36944	0.11	0.11	4.84
32	**RU**	0.30	5,055	61	34500	349	854	1780	3.44	243384	0.53	461264	0.17	0.07	1.10
33	**SE**	0.84	3,149	11	263	115	124		1.85	65632	2.16	65632	0.10	0.09	10.37
34	**SI**	0.59	982	2	40	29	35		2.29	39427	5.48	21591	0.07	0.06	8.20
35	**SK**	0.58	590	3	48	35	41		2.31	18614	0.96	19366	0.09	0.07	5.07
36	**TR**	0.25	1,813	2	341	2	2	80	3.80	6890	0.03	232935	1.00	1.00	0.78
37	**UA**	0.10	1,719	19	9168	94	94	330	6.53	55547	0.36	154415	0.20	0.20	1.11
38	**UK**	0.71	17,739	157	4412	2571	3000	2700	2.05	694617	3.40	613658	0.06	0.05	8.67

* adjusted for risk re-distribution of cases with unknown transmission risk

$$SSD = \frac{HIV_{svy}}{HIV_{pop}} \bullet \frac{N_{pop}}{N_{svy}}$$

Legend
- **questionable or unreliable values**
- **imputed values**
- **possibly unreliable value**
- **value likely too high**
- **value likely too low**
