# Supplementary material for: Estimating the size of the MSM populations for 38 European countries by calculating the survey-surveillance discrepancies (SSD) between self-reported new HIV diagnoses from the European MSM internet survey (EMIS) and surveillance-reported HIV diagnoses among MSM in 2009
Source: BMC Public Health. 2013 Oct 3;13:919. doi: 10.1186/1471-2458-13-919 (PMC3850943; doi:10.1186/1471-2458-13-919)
Supplement: Additional file 2 — Determining the empirical association between household internet access levels and survey-surveillance discrepancies. [file 1471-2458-13-919-S2.pdf]

Determining the empirical association between household internet access levels and survey-surveillance discrepancies between self-reported HIV diagnosis among MSM participating in MSM internet surveys and surveillance-system reported HIV diagnosis among MSM

	Proportion of households with broadband Internet access (%)					observed SSD				
year	Germany	France	UK	Switzerland	Netherlands	Germany	France	UK	Switzerland	Netherlands
2003	9		11		20	6.8				
2004	18	15	16	25	31		5.4		7.2	
2005	23		32		54			2.9		
2006	34	30	44	53	66	2.8	1.3	2.6		n.d.
2007	50	43	57	63	74	2.5			4.1	n.d.
2008	55	57	62	71	74			4.7		n.d.
2009	65	57	69	75	77		1.5		3.4	n.d.
2010	75	67	77	77	80	2.4	1.7	1.8	2.1	2.7
2011	78	70	83		83					

household internet access level (proportion in %)	observed/ constructed SSD	constructed from	discarded
9%	6.8		
15%	6.3	5.4 - 7.2	
30%			1.3
32%	2.9		
34%	2.8		
44%	2.6		
50%	2.5		
57%			1.5
63%			4.1; 4.7
66%	2.4	1.7 -1.7 -3.8	
67%			
75%	2.1	1.8 -2.1- 2.4	3.4
77%			
80%			2.7
